# Systemic Inflammation and Tumour-Infiltrating T-Cell Receptor Repertoire Diversity Are Predictive of Clinical Outcome in High-Grade B-Cell Lymphoma with *MYC* and *BCL2* and/or *BCL6* Rearrangements

**DOI:** 10.3390/cancers13040887

**Published:** 2021-02-20

**Authors:** Vito Olschewski, Hanno M. Witte, Veronica Bernard, Konrad Steinestel, Wolfgang Peter, Hartmut Merz, Johannes Rieken, Harald Biersack, Nikolas von Bubnoff, Alfred C. Feller, Niklas Gebauer

**Affiliations:** 1 Department of Haematology and Oncology, University Hospital of Schleswig-Holstein, Campus Lübeck, Ratzeburger Allee 160, 23538 Lübeck, Germany; vito.ol@gmx.de (V.O.); hanno.witte@uksh.de (H.M.W.); jojorieken@googlemail.com (J.R.); harald.biersack@klinikum-kulmbach.de (H.B.); nikolas.vonbubnoff@uksh.de (N.v.B.); 2 Department of Hematology and Oncology, Federal Armed Forces Hospital Ulm, Oberer Eselsberg 40, 89081 Ulm, Germany; 3 Institute of Pathology and Molecular Pathology, Federal Armed Forces Hospital of Ulm, Oberer Eselsberg 40, 89081 Ulm, Germany; konradsteinestel@bundeswehr.org; 4 Hämatopathologie Lübeck, Reference Center for Lymph Node Pathology and Hematopathology, 23562 Lübeck, Germany; bernard@haematopathologie-luebeck.de (V.B.); merz@haematopathologie-luebeck.de (H.M.); feller@haematopathologie-luebeck.de (A.C.F.); 5 HLA Typing Laboratory of the Stefan-Morsch-Foundation, 55765 Birkenfeld, Germany; wolfgang.peter@stefan-morsch-stiftung.de; 6 Institute for Transfusion Medicine, University Hospital of Cologne, Kerpenerstr 62, 50937 Köln, Germany

**Keywords:** immune responses to cancer, lymphoma, antibody immunotherapy

## Abstract

**Simple Summary:**

The current version of the World-Health-Organization (WHO) classification of tumors of hematopoietic and lymphoid tissues acknowledges the provisional entity of high-grade B-cell lymphoma, with *MYC* and *BCL2* and/or *BCL6* rearrangements (HGBL-DH/TH) which is associated with dire prognosis compared to triple-negative diffuse-large-B-cell-lymphoma (tnDLBCL). There is growing evidence for the essential prognostic role of the tumor-microenvironment (TME) and especially the extent of tumor-infiltration by the adaptive immune-system through tumor-infiltrating-lymphocytes (TIL) across a variety of cancers. More precisely, the clonal-architecture of the tumor-infiltrating T-cell-receptor (TCR)-repertoire has recently emerged as a key determinant of risk-stratification in patients with hematological malignancies. Moreover, inflammation-based prognostic-scores, such as the Glasgow-prognostic-score (GPS) were shown to reflect the TME. We therefore performed a large scale next-generation-sequencing (NGS) and clinicopathological study of the TCR-β-chain-repertoire in HGBL-DH/TH revealing several entity-exclusive clonotypes distinct from tnDLBCL, suggestive of tumor-neoantigen-selection and correlate our findings with the GPS in context of clinical outcome in HGBL-DH/TH.

**Abstract:**

High-grade B-cell lymphoma, with *MYC* and *BCL2* and/or *BCL6* rearrangements (double/triple-hit high grade B-cell lymphoma, HGBL-DH/TH) constitutes a provisional entity among B-cell malignancies with an aggressive behavior and dire prognosis. While evidence for the essential prognostic role of the composition of the tumor-microenvironment (TME) in hematologic malignancies is growing, its prognostic impact in HGBL-DH/TH remains unknown. In this study, we outline the adaptive immune response in a cohort of 47 HGBL-DH/TH and 27 triple-negative diffuse large B-cell lymphoma (tnDLBCL) patients in a large-scale, next-generation sequencing (NGS) investigation of the T-cell receptor (TCR) β-chain repertoire and supplement our findings with data on the Glasgow-Prognostic Score (GPS) at diagnosis, as a score-derived measure of systemic inflammation. We supplement these studies with an immunophenotypic investigation of the TME. Our findings demonstrate that the clonal architecture of the TCR repertoire of HGBL-DH/TH differs significantly from tnDLBCL. Moreover, several entity-exclusive clonotypes, suggestive of tumor-neoantigen selection are identified. Additionally, both productive clonality and percentage of maximum frequency clone as measures of TCR repertoire diversity and tumor-directed activity of the adaptive immune system had significant impact on overall survival (OS; productive clonality: *p* = 0.0273; HR: 2.839; CI: 1.124–7.169; maximum productive frequency: *p* = 0.0307; HR: 2.167; CI: 1.074–4.370) but not PFS (productive clonality: *p* = 0.4459; maximum productive frequency: *p* = 0.5567) in HGBL-DH/TH patients, while GPS was a significant predictor of both OS and PFS (OS: *p* < 0.0001; PFS: *p* = 0.0002). Subsequent multivariate analysis revealed GPS and the revised international prognostic index (R-IPI) to be the only prognosticators holding significant impact for OS (GPS: *p* = 0.038; R-IPI: *p* = 0.006) and PFS (GPS: *p* = 0.029; R-IPI: *p* = 0.006) in HGBL-DH/TH. Through the identification of expanded, recurrent and entity-exclusive TCR-clonotypes we provide indications for a distinct subset of tumor-neoantigenic elements exclusively shared among HGBL-DH/TH. Further, we demonstrate an adverse prognostic role for both systemic inflammation and uniform adaptive immune response.

## 1. Introduction

The recently revised World Health Organization (WHO) classification of tumors of hematopoietic and lymphoid tissues now acknowledges the new and provisional entity of high-grade B-cell lymphoma, with *MYC* and *BCL2* and/or *BCL6* rearrangements (HGBL-DH/TH) [1]. Special emphasis was laid on this group of aggressive B-cell lymphomas in recognition of its complicated classification due to the concurrence of features of both Burkitt lymphoma (BL) and diffuse large B-cell lymphoma (DLBCL) including cytogenetic aberrations affecting *MYC*, *BCL2* and/or *BCL6* [2]. From a clinical perspective, HGBL-DH/TH pose an unresolved and troublesome issue, as they recurrently exhibit adverse prognostic features including advanced stage disease with higher revised international prognostic index (R-IPI) at diagnosis and a high rate of bone marrow as well as central nervous system (CNS) involvement, while outcome in patients treated according to conventional immunochemotherapeutic regimens such as R-CHOP (rituximab, cyclophosphamide, doxorubicin, vincristine and prednisone) with or without consolidating high-dose chemotherapy regimens followed by stem-cell transplantation remains grim [3,4].

Numerous studies underline the essential impact of the tumor-microenvironmental (TME) composition on clinical outcome of cancer patients across a variety of entities [5,6,7,8]. Amongst the plethora of immune cells, especially the tumor-infiltrating-lymphocytes (TILs), which represent adaptive immunity, have become a subject of interest. The interplay between immune cells and tumor cells is increasingly important. For a variety of malignancies, an association between an increased T-cell infiltration and a favorable clinical outcome has been demonstrated [7]. T-cell receptors (TCR) determine the diversity of T-cells. The most variable component of TCRs is represented by the complimentary determining region 3 (CDR3) of the TCRβ chain. The role of the TCRβ CDR3 sequence for MHC (major histocompatibility complex) -peptide mediated recognition of T-cells is crucial [9]. Therefore, the TCRβ CDR3 sequence serves as a reliable molecular marker for individual T-cell clones. The range of T-cell clones and their individual TCR repertoire determine TCR diversity and frequency. An association between increased T-cell infiltration and a favorable outcome in patients with both DLBCL as well as solid tumors has been reported in previous studies [5,6,7]. Our group recently established this coherence in sporadic Burkitt lymphoma [10].

A restricted TCR repertoire was recurrently associated with an adverse prognostic impact [5,11]. To date, the impact of the TCR repertoire regarding the adverse clinical course of HGBL-DH/TH compared to DLBCL remains unknown.

Simultaneously, systemic inflammation has an impact on the phenotype of cellular components of the tumor microenvironment and affects levels of cytokines. Proinflammatory mediators and tumor-infiltrating immune cells regulate serum levels of C-reactive protein (CRP). Albumin levels also correlate with inflammatory responses. Prognostic scores based on primarily innate inflammatory activity, assessed through metabolic and immunological parameters of the peripheral blood, have proven valuable for risk stratification across a variety of malignancies, including DLBCL, and were shown to reflect the TME [12]. Apart from inflammation, different staging systems and risk scores contain markers of nutritional state, such as albumin serum levels at initial diagnosis. One such score which represents inflammation as well as nutritional status is the Glasgow Prognostic Score (GPS). The GPS differentiates three different subgroups (group I: 0 points; group II: 1 point; and group III: 2 points) by calculating one point for CRP value of >10 mg/dL and another point for serum albumin of <35 g/L.

Here we present a large-scale next-generation sequencing (NGS) study of the CDR3 region of the TCR β-chain repertoire in a cohort of HGBL-DH/TH, while including a comparative analysis with a previously reported cohort of DLBCL showing no aberrations in *BCL2*, *BCL6* and *MYC* (tnDLBCL) [10]. Moreover, we correlate the established and validated inflammation-based Glasgow-prognostic score (GPS) with TCR repertoire diversity in the context of clinical outcome in this rare type of lymphoma.

## 2. Results

### 2.1. Baseline Clinical Characteristics

Median age in the current study population (N = 74; HGBL-DH/TH n = 47; tnDLBCL n = 27) was 71.5 years (range, 18–89 years) while 54.1% were male. Age distribution in HGBL patients and DLBCL patients was comparable (*p* = 0.1379). Median follow-up period was 30.5 months (1–115 months; 25% percentile 17.3; 75% percentile, 51.7). R-IPI of 0 was present in two patients (2.7%) while a R-IPI of 1–2 could be observed in 33 (44.6%) patients and a R-IPI of >2 in 39 (52.7%) patients of the study group. Extranodal disease at initial diagnosis was detected in 51 cases (68.9%). Six patients (8.1%) had an ECOG performance status >2 at initial clinical presentation. Baseline staging identified 43 patients (58.1%) with Ann-Arbor stage III/IV. Thirty-eight patients (51.4%) showed B-symptoms at initial diagnosis. There were no significant distinctions for each component of the R-IPI between HGBL patients compared to DLBCL patients. Detailed baseline clinical characteristics are depicted in Table 1.

### 2.2. Laboratory Assessment

Median LDH serum level was 412 U/l (range, 113–14,638 U/L). Elevation of LDH levels at initial diagnosis could be assessed in 48 cases (64.9%). Laboratory analysis revealed a median albumin value of 37.3 g/L for the whole study population (range, 21.7–54.3 g/L). Median C-reactive protein (CRP) in the overall study group was 11.1 mg/dL (range, 1.0–44.4 mg/dL). A GPS of 0 was calculated for 13 HGBL-DH/TH patients (27.7%) and 17 patients with tnDLBCL (63.0%). In 16 patients of the study cohort (21.6%) a GPS of 1 was assessed (9 HGBL-DH/TH patients and 7 patients with tnDLBCL) while a GPS of 2 could be detected in 28 patients (37.8%; 25 HGBL-DH/TH patients and 3 tnDLBCL patients).

### 2.3. Treatment Modalities

As first line treatment, a R-CHOP-like regimen was conducted in the majority of cases (55/74 cases, 74.3%). 14 patients with HGBL-DH/TH and two patients with tnDLBCL received other therapeutic approaches. The overall-response rate (ORR) in the study was 82.4%. Complete responses (CR) could be achieved in 16 patients suffering from HGBL-DH/TH (34.0%) and 14 patients with tnDLBCL (51.9%) while partial remissions (PR) were depicted in 20 HGBL-DH/TH patients (42.6%) and 11 patients with tnDLBCL (40.7%). Progressive disease (PD) was captured for nine (12.2%) patients (seven HGBL-DH/TH cases and two tnDLBCL cases). Therapeutic intensification in terms of autologous or allogenic hematopoietic stem cell transplantation (autoHSCT/alloHSCT) was performed in six (three autoHSCT and three alloHSCT) cases of HGBL-DH/TH (12.8%) in further course of the disease. The majority of autoHSCT or alloHSCT was required in relapsed or refractory setting (five cases; 83.3% of cases undergoing HSCT). There were two patients (2.7%) refusing further treatment options.

Relapse rate in the HGBL-DH/TH subgroup was 57.4% (21 relapses in HGBL-DH cases and six HGBL-TH cases) and 14.8% in tnDLBCL patients (n = 4), respectively.

Severe adverse events due to cytoreductive first line treatment could be detected in 51 patients (68.9%). Toxicity profile was moderate and predominantly hematological (n = 27) as well as infectious (n = 23) in nature. Nearly half (43.5%; 10/23) of infectious adverse events occurred in patients for which a GPS of 2 was calculated. Detailed depiction of the toxicity profile is outlined in Table 2.

### 2.4. Histopathological Assessment, Immunophenotyping and FisH

Upon systematic assessment of the TME from a morphological and immunohistochemical perspective employing a standardized panel of antibodies, we discovered a wide variety in the density of T-cell infiltrates in HGBL-DH/TH samples. These ranged from loosely interspersed cells to focal dense infiltrates. Upon histopathological reassessment, we evaluated a median tumor cell content of 85%.

Immunohistochemical data on BCL2, BCL6 and MYC expression as well as cell of origin (COO) as determined immunohistochemically via the well-established algorithm by Hans et al. showing a germinal center type expression pattern (GCB-type) in 42 cases (56.8%) and a non-germinal center type expression pattern (non-GCB-type) in 32 cases (43.2%) are briefly summarized in Table 3 [13].

**Figure 1:**
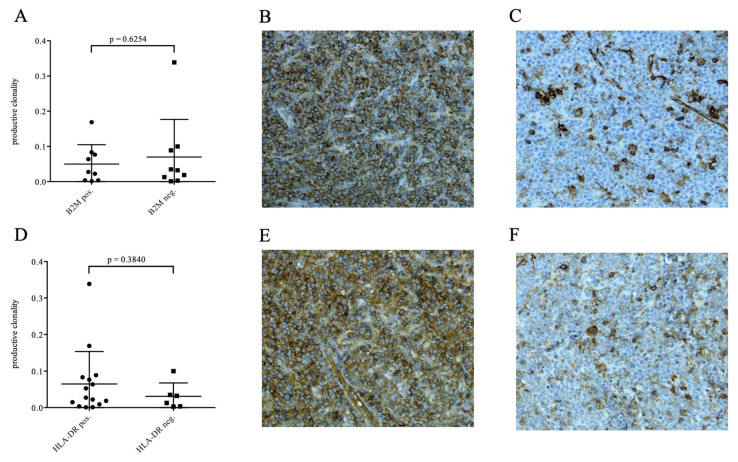
Immunohistochemical expression of HLA class I antigen compound beta2microglobulin was observed to be lost in a substantial subgroup of HGBL DH/TH patients (**B**,**C**) yet shows no significant impact on TIL TCR clonality measures, signified by productive clonality. Similar results were observed regarding other clonality measures (**A**); %top10 clones, max. productive frequency; data not shown). Moreover, while immunohistochemical loss of expression of HLA class II antigen HLA-DR was less prevalent (**E**,**F**), again, o significant impact on clonality measured could be observed (**D**).

Moreover, as loss of both human leukocyte antigen (HLA) class I and II expression is a common phenomenon in aggressive B-cell lymphoma pathogenesis, yet TCR-mediated anti-tumor activity is widely dependent on an intact HLA apparatus, we correlated our observations regarding TCR clonality measures with immunohistochemical expression of HLA class I antigen beta-2-microglobulin (B2M) and HLA class II antigen HLA-DR in all cases in which sufficient FFPE tissue was still available following molecular assessment (18 and 21 respectively). We observed a substantial fraction of cases with loss of B2M and/or HLA-DR yet could not identify an impact on clonality measures (Figure 1).

FISH studies revealed 47 cases of HGBL-DH/TH of which 40 cases were HGBL-DH and seven cases were identified as HGBL-TH. Moreover, we identified 27 cases of tnDLBCL as a reference group.

Epstein-Barr encoding region (EBER) in-situ hybridization was found to be positive in 10 cases of tnDLBCL (EBV-positive DLBCL) while there was no detection of EBV-positive cases in the HGBL-DH/TH cohort. In order to compensate for the above average frequency of EBV-positive cases in our tnDLBCL cohort, we randomly excluded five EBV positive cases and tested for shifts in statistical significance. Despite performing this compensation in independent triplicates, results were not altered significantly, as suggested by the non-significant impact of EBV-status in tnDLBCL. Intriguingly, in a comparative analysis of EBV positive DLBCL, NOS and tnDLBCL, there were no significant differential findings regarding any of the assessed clonality measures.

An impact of site of origin on TIL TCR repertoire clonality could neither be detected in a pooled nor in a stratified analysis according to cytogenetic subtype (data not shown).

### 2.5. High Throughput TCR Sequencing

Next-generation sequencing of the CDR3 region of the TCRβ chain was performed on 65 samples from all 47 patients with HGBL-DH/TH harboring *MYC* and *BCL2* and/or *BCL6* rearrangements and 27 tnDLBCL samples. Further analysis was restricted to samples with a sufficient number of productive TCRs (>100 productive TCRs/sample) in order to avoid the detection of pseudo-clonality in tissues exhibiting an artificially low T-cell count.

**Figure 2:**
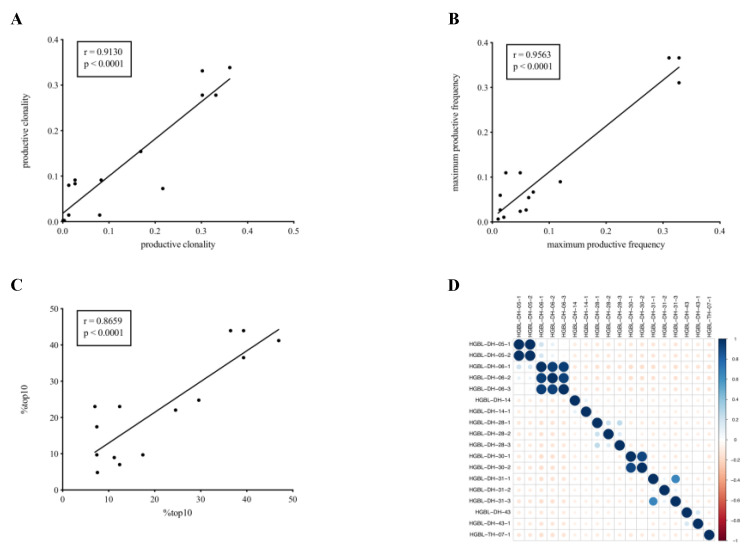
Comparative analysis of multiple samples of a given patient. When analyzing two samples from a given patient derived from separate locations at the same point in time, we found a high degree of correlation according to Pearson’s-correlation with regard to productive-clonality (**A**), maximum-frequency of a productive-clonotype (**B**), percentage of the frequency of the %top10 most expanded TCR-clonotypes (**C**) as well as TCR-sequence overlap as illustrated by a linear heat map of sequence homology (**D**).

Among the group of HGBL-DH/TH samples subjected to further analysis a median of 1504.5 (range 136–24,680) rearrangements was sequenced. In patients where multiple samples were available for analysis, all of which passed quality control measures (HGBL-DH/TH and tnDLBCL) (n = 27) we detected a highly significant correlation between samples in terms of our previously defined clonality measures as well as regarding sequence homology (Figure 2A–D).

**Figure 3:**
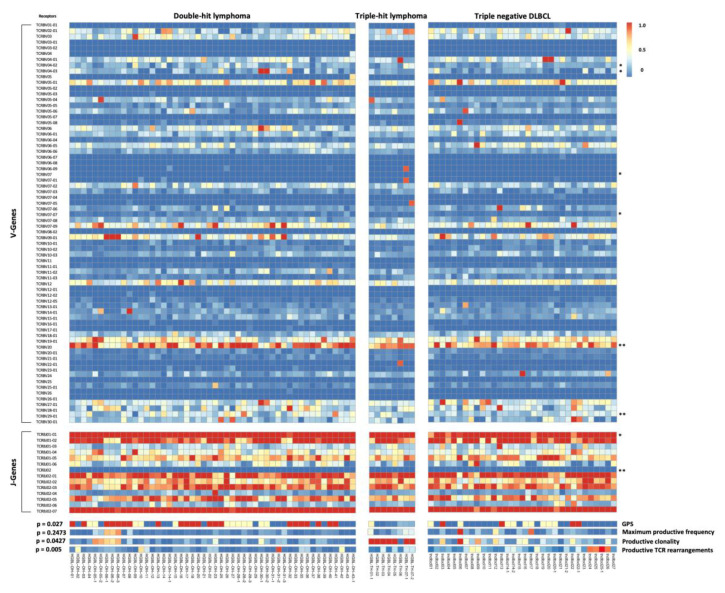
The usage of Vβ- and Jβ-genes in tumor samples from HGBL DH/TH samples as well as biopsy specimen acquired at relapse compared with tnDLBCL Heatmap of the usage frequency. For several Vβ- and Jβ-segments a significant differential usage between pooled HGBL-DH/TH-samples at diagnosis and tnDLBCL samples was observed and was subsequently marked with * *p* < 0.07 (results bordering on statistical significance) and ** *p* < 0.05 (significance level) according to two-tailed t-test.

Overall 155,658 individual clonotypes were identified, most of which were only present at minimal frequencies in a minor fraction of cases. Figure 3 demonstrates the usage frequencies of TCR Vb and Jb gene in each sample. The usage frequency of TCR Vβ- and Jβ-genes are shown to differ (borderline) significantly with regard to the recombination of several Vb- and one Jb-gene segments.

### 2.6. Comparison of the TCR Repertoire between HGBL and DLBCL

**Figure 4:**
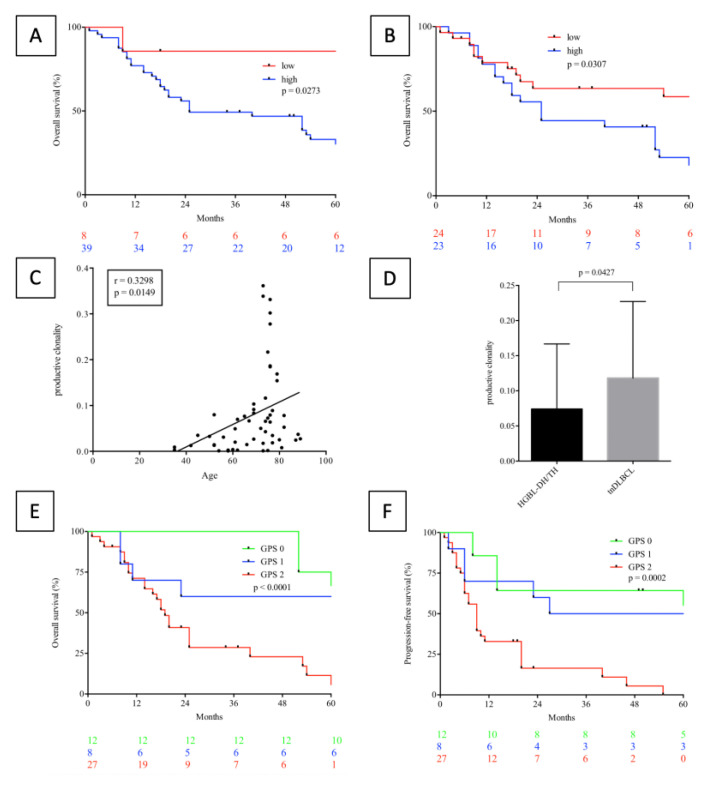
Clinical impact of the TCR repertoire clonality in HGBL-DH/TH. Overall survival (OS) (**A**,**B**) of HGBL-DH/TH patients stratified according to productive TCR clonality (**A**) and maximum productive frequency (**B**) displays significant prognostic capabilities of the clonal architecture of the tumour-infiltrating TCR repertoire in HGBL-DH/TH with regard to OS but not progression free survival (PFS, data not shown, Supplementary Figure S1) (productive clonality: *p* = 0.0273; HR: 2.839; CI: 1.124–7.169; maximum productive frequency: *p* = 0.0307; HR: 2.167; CI: 1.074–4.370). Productive clonality in HGBL-DH/TH patients is a statistically significant function of age (*p* = 0.0149; r = 0.3298); (**C**) while a similar trend of borderline statistical significance was observed for maximum productive frequency (*p* = 0.0599; r = 0.2577; Supplementary Figure S1). Upon comparative analysis of clonality measures between HGBL-DH/TH and tnDLBCL, a significant difference in terms of productive clonality was observed (*p* = 0.0427), (**D**). Further an impact of systemic innate immunity mediated inflammation, according to the Glasgow prognostic score, opposing the prognostic implications of an elevated degree of adaptive intra-tumour immune response was observed for both OS (*p* < 0.0001), (**E**) and PFS (*p* = 0.0002), (**F**).

Comparison of the TCR repertoire analysis between HGBL-DH/TL and tnDLBCL is outlined in Table 4. Upon comparative analysis of HGBL-DH/TH and tnDLBCL there was a significant distinction regarding productive TCR rearrangements between HGBL-DH/TH and tnDLBCL (HGBL-DH/TH: median 1504.5, 136–24,680; tnDLBCL: median 3447.0, range 1642–1745; *p* = 0.005). This value measures the total number of functional T-cells infiltrating the tumor. Current results further revealed a narrower TCR repertoire for tnDLBCL (productive clonality: median 0.036, range 0.0009–0.3614 for HGBL-DH/TH versus median 0.08785, range 0.0013–0.4437 for tnDLBCL, *p* = 0.0427) (Figure 4D). There was no detection of a significant differentiation regarding the analysis of the “% maximum frequency clone” in HGBL-DH/TH compared with tnDLBCL (*p* = 0.2473). This measure represents a quantitative analysis of the most highly expanded clones.

Moreover, we encountered fourteen public (mean frequency ≥ 0.2%) TCRβ CDR3 clonotypes exclusively yet recurrently found in HGBL-DH/TH (n ≥ 3) not present in tnDLBCL (Table 5). Most of these clones were detectable at relatively high frequencies (median 0.407%; range 0.205–1.822%) indicating biological significance and potential tumor neoantigenic selection. We screened these expanded sequences by cross comparison with the most comprehensive publically available dataset of healthy controls and patients with defined medical conditions, revealing previously non-described clonotypes in all but one sequence, which was shown to target EBNA3A, an integral nuclear Epstein-Barr virus-derived protein [14]. This specific clonotype was present in three cases of HGBL-DH/TH while none of them was found to be EBV-associated.

### 2.7. The Impact of TCR-Diversity and Inflammation on Survival Outcome

As demonstrated by log-rank testing and Kaplan-Meier analysis both elevation of productive clonality and the percentage of maximum frequency clone as measures of TCR repertoire diversity and tumor-directed activity of the adaptive immune system had significant adverse impact on OS (productive clonality: *p* = 0.0273; HR: 2.839; CI: 1.124–7.169; maximum productive frequency: *p* = 0.0307; HR: 2.167; CI: 1.074–4.370) but not PFS (productive clonality: *p* = 0.4459; maximum productive frequency: *p* = 0.5567) if dichotomized as described [15] (Figure 4A,B, Supplementary Figure S1). Similar to results recently obtained by Simnica et al. we further found these parameters to be a function of age (Figure 4C and Supplementary Figure S1). Intriguingly, these parameters, reflecting the clonal architecture of the tumor-infiltrating TCR repertoire, differ from DLBCL, independently from age (Figure 4D and Supplementary Figure S1) [16]. Contrastingly, an elevated GPS as an indicator of undirected innate immunological response and inflammation was shown to significantly predict OS and PFS (Figure 4E,F). Subsequent multivariate analysis revealed GPS (*p* = 0.029) and R-IPI (*p* = 0.006) to be the only prognosticators holding significant impact for OS while clonality measures were just bordering on statistical significance (*p* = 0.082 for maximum productive frequency and *p* = 0.294 for productive clonality). Current data detected a significant higher rate of productive TCR rearrangements in patients with aggressive B-cell lymphomas with a GPS of 0 compared to a GPS of 1 (*p* = 0.0415) or a GPS of 2 (*p* = 0.0227) (Supplementary Figure S2).

## 3. Discussion

We performed large-scale NGS study of the TCR β chain repertoire of HGBL-DH/TH. The aim of the study was to demonstrate the potential prognostic implications of the TCR repertoire in tumor-infiltrating T lymphocytes in HGBL-DH/TH. These findings are, to the best of our knowledge, the first description of prognostic implications associated with systemic inflammation and local adaptive immune response in HGBL-DH/TH, especially emphasizing the TCR repertoire as part of the TME.

The robustness of immunosequencing of genomic DNA derived from FFPE samples constitutes a substantial advantage of this method. In contrast to single-cell sorting approaches or limitation on transcribed variants in RNAseq-approaches, there is independence from variability for immunosequencing methods [11,17,18]. However, further studies should include comparative RNAseq data from fresh-frozen tissue samples in order to allow focus on expressed TCR-subsets.

Regarding the high frequency of the GCB-subtype in HGBL-DH/TH, the current results are in line with the previously published large scale study (n = 2.439) by Rosenwald et al. in which HGBL-DH/TH that involved *BCL2* rearrangements exclusively fell into the GCB subgroup, whereas those that involved *BCL6* were found in both the GCB as well as the non-GCB subgroup [19]. Another study by Sha et al. confirmed these results by detecting 75% of GCB-subtypes in HGBL-DH/TH cases unlike double-expressor lymphoma, where indeed a high frequency of post-germinal center B-cell phenotype lymphomas are encountered [20]. Considering the high frequency of non-GCB subtypes in the cohort of tnDLBCL patients, different studies associate the non-GCB subtype with adverse prognosis compared with the GCB subtype [19,21]. Therefore, the current study comprises two homogenous subgroups of different aggressive B-cell lymphoma subtypes harbouring relevant adverse prognosticators for comparative analysis.

For a variety of cancer subtypes it has been shown that an activated and multilayered TME can be associated with a relevant prognostic impact on clinical outcome [22,23]. In accordance with these findings Keane et al. demonstrated that the TCR repertoire in B-cell lymphomas represents a key determinant of the TME [11].

Our current high-throughput immunosequencing-data reveal a more diverse TCR repertoire for HGBL-DH/TH compared with tnDLBCL while a higher number of functional T-cells could be detected in tnDLBCL (productive TCR rearrangements). Moreover, there were no significant distinctions regarding the level of T-cell infiltration as well as the maximum productive frequency of the tumor infiltrating T-cells between the two entities. In keeping with results from a large-scale sequencing approach by Simnica et al., which recently found human TCR-repertoire metrics to systematically deteriorate in the aging individual, immunological fitness which is represented by TCR productive clonality and other measures can be interpreted as a function of age [16]. Within the current study cohort, the distribution of age in HGBL-DH/TH and tnDLBCL patients was comparable while age-dependent measures of productive clonality differed significantly between these subgroups. The constellation of findings from immunosequencing for tnDLBCL were comparable to results from TCR repertoire analysis recently reported by Keane et al. [11].

In a prior study, we found no association between the restriction of HLA-DR and the impact on tumour-infiltrating T-cells and its impact on survival appears to be limited to the pre-rituximab era [24]. This is supported by Tada et al. who found that HLA-restriction has no significant impact on clinical outcome in aggressive B-cell lymphomas in the rituximab era [25]. As loss of both HLA class I and II expression constitutes a recurrent driver in aggressive B-cell lymphoma pathogenesis and TCR-mediated anti-tumor activity requires an intact HLA apparatus, we were intrigued to observe a substantial subgroup of patients harboring an immunohistochemical loss of B2M and/or HLA-DR which did however not impact clonality measures (Figure 1A–E) [26,27]. However, future studies should integrate an even more comprehensive HLA-typing and immunohistochemical assessment of the HLA class I and II apparatus for analyzing the impact of a potential underlying HLA restriction on the occurrence of specific TCR-sequences as well as clonality measures.

Findings of a more diverse TCR repertoire can be interpreted as an inadequate adaptive immune response which is negatively influenced by malignant B-cells. Recent reports suggest that a higher clonality of tumor infiltrating T-cells can be associated with a more specific and effective immune response. Consistent with this assumption, data from solid tumors revealed poor clinical outcomes caused by CTLA-4 blockade resulting in a diversification of the TCR repertoire while PD-1 blockade lead to a higher clonality of the TCR repertoire and a consecutive more favorable clinical outcome [28,29]. Possibly, a higher percentage of maximal clone frequency can be associated with a higher responsiveness to anti-PD-1 therapeutic agents [30]. Since no patient included in the current study received immunotherapeutic checkpoint inhibition, this notion should be validated in prospective clinical trials. Apart from therapeutic checkpoint blockade, the present data demonstrate that highly expanded TCR-clonotypes can be associated with adverse prognosis in HGBL-DH/TH.

Upon univariate analysis the current results suggest that clonality measures in terms of a restricted TCR-repertoire seem to determine inferiority of clinical outcome (OS but not PFS) in HGBL-DH/TH patients. This finding failed to hold its independent prognostic capability upon subsequent multivariate Cox proportional hazard calculation. Different features and components of the TME must be incorporated regarding the evaluation of the functionality of the TCR repertoire and its immunosurveillance.

Comparing the diversity of the TCR repertoire and productive TCR rearrangements between HGBL-DH/TH and tnDLBCL, findings from the present study lead to the conclusion that immunotherapy approaches might be more encouraging in tnDLBCL patients [11]. Concurrently, productive clonality which influences response to immunotherapy differed significantly between HGBL-DH/TH and tnDLBCL (*p* = 0.0427). Accordingly, a higher diversity of the TCR-repertoire decreases the probability of immunologic tumor antigen escape [31].

However, fourteen public clonotypes could be identified in HGBL-DH/TH harboring the potential for tumor neoantigenic selection. Contingently, these subdominant T-cell clones are able to target tumor neo-antigens [32]. It is tempting to speculate, that these clonotypes might constitute immunologic responses which are directly associated with characteristic cytogenetic aberrations in HGBL-DH/TH. Our study group recently reported on concomitant results in Burkitt-lymphoma suggesting an equivalent conclusion [10]. Moreover, targeted sequencing approaches in HGBL-DH/TH revealed a higher grade of complexity regarding tumor mutational burden and tumor neo-antigen burden compared to tnDLBCL [33].

The finding of distinctive public clonotypes in HGBL-DH/TH is in keeping with the results published by Linnemann et al. suggesting a direct correlation between neoantigen-specific T-lymphocytes and mutational load in the tumor tissue [34]. Therefore, several publications suggest that T-cell diversity in tumor tissue vary from those in the peripheral blood [35]. On the contrary, the recent work by Wu et al. delineated the detection of expanded TCR clonotypes in tumor tissue, normal adjacent tissue as well as in the peripheral blood [36]. Conclusively, tumor neo-antigens should be identified in an independent and extended cohort by whole exome sequencing (WES) combined with whole transcriptome sequencing as well as proteomic analysis. Furthermore, the performance of these functional assays including peripheral blood samples will elucidate the debate on the distinctiveness of TCR-clonotypes in HGBL-DH/TH compared to tnDLBCL.

In order to functionally validate our observations and to associate our major HGBL-DH/TH-exclusive clonotypes with their targeted epitopes, we aim to supplement our DNA-based TCR sequencing approach with single-cell RNA-sequencing as was recently described [37]. Further, we hope, that through the application of a recently described epitope identification approach for TCR sequences employing cytokine-capturing antigen-presenting cells, we might uncover the antigenic elements targeted by the adaptive immune system in our HGBL-DH/TH cohort [38]. An alternative concept might be the lentiviral delivery of libraries of potential epitopes into cells for endogenous processing and presentation on major histocompatibility complex (MHC) molecules employing T-scan, as described [39].

Even though we were able to identify one specific T-cell receptor β CDR3 clonotype for the molecular recognition of the integral nuclear EBV-derived protein EBNA3A in HGBL-DH/TH, none of these cases of HGBL-DH/TH could be associated with an underlying EBV infection by EBER in-situ hybridization. This may well be attributed to non-tumor specific T-cell infiltrates. Unfortunately, no information on EBV serology was available for comparative analysis. Additionally, our results need to be interpreted in the context of recent observations by Mundo et al. who recently reported on a significant proportion of B-cell lymphomas which were false negative for EBV by routine techniques [40].

There is growing evidence stating a drastic impact of GPS on outcome in various malignancies including lymphoma (e.g., DLBCL, cHL, multiple Myeloma) and solid tumors [41,42]. This is the first study to illustrate the prognostic impact of GPS and TCR clonality in HGBL-DH/TH.

Limitations of the present study include the size of the cohort, albeit one of the largest in this rare entity, and its retrospective design. Our results need to be validated in larger data sets, derived from prospective trials before definitive suggestions regarding personalized risk-assessment ultimately guiding potential treatment intensification should be made. Due to the rarity of HGBL-DH/TH, the retrospective character of the present study was inevitable. Hence, prospective blood sampling for comparative analysis between the peripheral blood and tumor tissue samples would have been preferable but unrealizable within this exploratory study approach. Clinical characteristics are derived from medical records, harboring the potential for fragmentary data.

While the CDR3 region is regarded as a robust outline of TCR clonality, it provides a restricted view on the TCR β chain repertoire. This is likely to have led to an underestimation of clonal diversity compared with the combined analysis of both TCR α- and β chain repertoires, which also may have led to an overestimation of shared TCRαβ clonotypes between patients, as the potential pairing of different TCRBV and TCRAV chains would have escaped detection in the current study. Moreover, our analyses do not consider MHC polymorphisms. A comprehensive analysis of gene expression patterns as well as immunohistochemical features of a wider spectrum concerning HLA-components would have been desirable but was beyond the scope of the current study and should be a subject for further investigations.

Our observations hold the potential to impact clinical practice as current efforts seek to identify patients in need of upfront therapeutic intensification. The need for refined risk-stratification at baseline and the investigation of the potential of adaptive immunity in this difficult-to-treat entity is therefore evident.

## 4. Materials and Methods

### 4.1. Samples

Drawing from our institutional cytogenetics database, we identified patients with complete information on *BCL2*, *BCL6* and *MYC* rearrangements. We then screened all consecutive HGBL-DH/TH samples sent to the Reference Centre for Hematopathology’s registry Lübeck (Prof. Feller) from 1 January 2008 to 31 July 2017 that fulfilled all diagnostic criteria in accordance with the 2016 revision of the WHO classification of tumors of hematopoietic and lymphoid tissues in the current study [1]. Diagnosis of HGBL-DH/TH was confirmed by two experienced hematopathologists.

Application of our internal quality measures resulted in the exclusion of 14/79 HGBL-DH/TH samples as a significant number of productive templates was not reached in these cases (n = 100).

Sixty-five formalin-fixed and paraffin-embedded (FFPE) samples at diagnosis from 47 patients could be included into the final analysis. Diagnostic samples from both nodal as well as extranodal primary manifestations were included.

While extranodal manifestations were present in a majority of cases, biopsy samples were recurrently taken from nodal lesions leading to samples taken from patients with extranodal disease being classified as “nodal”. In order to assess the impact of immunochemotherapy as well as the immunological constellation within the TME at relapse 7 samples from seven patients were assessed as part of a longitudinal approach (median sampling interval was 22 months). After the exclusion of two samples, we further examined 34 samples from 28 triple negative (fluorescence in situ hybridization (FisH) negative for aberrations at *MYC*, *BCL2* and *BCL6*) DLBCL patients (10 EBV positive, 24 EBV negative samples) for comparative investigations (Supplementary Table S1). Case selection was merely based on availability of consecutive biopsy specimen with fitting cytogenetics. Due to this coincidental composition of the cytogenetically defined control group, this subgroup includes 10 cases of EBV-positive DLBCL, NOS and was further significantly skewed towards HGBL-DH/TH. Comparability was given due to the exclusion of patients with suspected immunosuppressive diseases. All cases that matched the predefined diagnostic criteria and were validated by the hematopathology panel underwent subsequent clinical assessment.

### 4.2. Clinical Assessment

The cohort comprised 47 fully annotated patients with histologically as well as cytogenetically confirmed HGBL-DH/TH. Twenty-seven patients with *de novo* DLBCL were included for comparative analysis as described [10]. Patients with insufficient follow-up, primary central nervous system lymphoma (PCNSL), post-transplant lymphoproliferative disorder (PTLD), HIV-related lymphoma as well as transformed indolent lymphoma were excluded. Clinicopathological features of the study group are briefly summarized in Table 1. Clinical information was collected from the original files and comprised patients’ performance status (Eastern Cooperative Oncology Group [ECOG]), stage, treatment modalities, therapeutic response, pattern of relapse, baseline serum levels of lactate dehydrogenase (LDH), revised international prognostic index (R-IPI) [43] and information on survival were anonymously coded alongside hematopathological assessments and TCR sequencing results. Extent of disease was routinely evaluated according to the Cotswold modifications of the Ann Arbor classification [44]. Data collection also included the calculation of the inflammation-based GPS as a complementary resource of risk stratification. The GPS differentiates three different subgroups (0 points; 1 point and 2 points) by calculating one point for CRP value of ≥10 mg/dL and another point for serum albumin of ≤35 g/L. [45]. Following baseline staging, patients were treated with a chemotherapeutic regimen of the treating physician’s choice with the respective current DSHNHL/GLA recommendations at the time serving as institutional standard.

### 4.3. Immunophenotyping, Tumor Infiltrating T-Cell Quantification and FisH

Immunophenotypic analysis was performed through panel based immunohistochemical assessment on FFPE sections. Histological slides were reviewed by VO, ACF, HM and NG in order to characterize qualitative and quantitative aspects of tumor-infiltrating lymphocytes showing very high reproducibility (data not shown). Number of productive rearrangements but not productive clonality was shown to be a statistically significant function of quantitative T-cell infiltration of a given sample (Supplementary Figure S3). Antibodies and clones utilized in the study are summarized in Supplementary Table S2.

Chromosomal breakpoints were analyzed by means of FisH using dual-color break- apart probes for *MYC*, *BCL2* and *BCL6* (Abott Vysis, Des Plaines, IL, USA) according to the manufacturer’s instructions. The presence of the t(8;14)(q24;q34) and exclusion of additional genomic aberrations affecting *BCL2* and/or *BCL6* was a mandatory inclusion criterion.

### 4.4. High-Throughput Sequencing of the CDR3 Region of the TCRβ Chain

Following isolation of genomic DNA (Supplementary methods), next-generation immunosequencing of the CDR3 region of the TCRβ chain was performed employing the ImmunoSEQ-Assay (Adaptive Biotechnologies, Seattle, Washington, DC, USA) at a targeted depth of 30,000 reads per sample. A DNA quantity of 500 ng was defined as sufficient. Two genomic aliquots per sample (if possible up to 1.500 µg per sample) were separately amplified in a two-step multiplex PCR adding adaptor sequences containing sample-identifying barcodes. Correction for primer bias was performed as described [46]. First, by employing forward and reverse amplification primers specific for every V and J gene segment the hypervariable CDR3 region of the TCR locus was amplified. Secondly, barcode sequence and Illumina^®^ adapter sequences were ligated. Fragments of 87 bp covering the CDR3 region alongside the specific flanking V and J genes, resulting from the multi-step PCR, were sequenced on an Illumina^®^ MiSeq using version 3.0 chemistry and 23 samples per lane.

Raw sequencing data were demultiplexed according to the abovementioned proprietary barcode sequences. Data analysis continued by removing adapter and primer sequences from demultiplexed reads, primer dimer, germline and other contaminant sequences. Subsequently clustering was performed, employing a modified nearest-neighbor algorithm and the relative frequency ratio between similar clones, to correct for technical errors introduced through multi-step PCR and sequencing. These revised sequences were of sufficient specificity to allow annotation of the V(N)D(N)J genes in accordance with the IMGT database (www.imgt.org accession date 27 November 2020) as “total productive sequences”. Raw sequencing run folders were subsequently uploaded to Adaptive Biotechnologies and processed through the in-house pipeline. Results were then further processed via the Adaptive Biotechnologies Analyzer 3.0.

### 4.5. TCR-Sequencing Data Analysis

As described, CDR3 sequences were normalized and quantified against a set of synthetic TCR Beta CDR3 templates, to correct for amplification bias [46].

Shannon’s entropy was used to calculate TCR repertoire entropy based on the frequency of particular sequences (Supplemental methods). The diversity of the distribution of CDR3 clones rises with increasing values for entropy [47]. On the contrary, clonality, which is the reciprocal of the normalized entropy, represents the uniformity of the distribution of TCR clones independent of sample size.

In order to further characterize the degree of expansion of selected clones in our cohorts the “% maximal frequency clone” calculation was performed, describing the relative frequency of the most prominent clone compared with the cumulative frequency of all clones [11]. The percentage frequency of the top10 dominant clones was determined in each sample. Accordingly, the measurement of the relative frequency of the ten most expanded clones in comparison with the cumulative frequency of all clones, resulted in frequency values normalized for the percentage of each clone in a sample enabling us to compare samples regardless of the individually achieved sequencing depth.

### 4.6. Comparative Analysis with DLBCL

All clonotypes meeting predefined criteria of expansion were considered to be suggestive of a shared tumor-neoantigen selection. These were subsequently investigated in a large publically available dataset comprised of healthy donors as well as a wide range of patients with various disorders (VDJdb) and were annotated accordingly (Supplementary methods) [48]. Clonotype comparative analysis was carried out for HGBL-DH/TH (n = 65) and tnDLBCL samples (n = 34). For details on tnDLBCL reference group see Supplementary Table S1.

### 4.7. Statistical Analysis

Differences among patient subgroups were assessed employing the chi-square as well as the (paired) two-tailed Student’s t-test, respectively. Correlation of continuous variables was assessed employing the Pearson approach. Progression-free and overall survival (PFS, OS) were calculated from the date of initial diagnosis. Survival (PFS and OS) was initially estimated by means of the Kaplan–Meier method and univariate log-rank test. Characteristics with significant impact on either OS or PFS were subjected to a subsequent multivariate proportional hazard analysis. All statistical investigations were conducted using Excel (Microsoft, Seattle, WA, USA), PRISM 6 (Graph Pad Inc., San Diego, CA, USA), R-Studio (Boston, MA, USA) and SPSS 25 (IBM, Armonk, NY, USA). Optimal cut-offs for survival analysis were determined employing and AUC-based algorithm proposed by Budczies et al. (productive clonality: 0.0033; maximum productive frequency: 0.0212) [15].

### 4.8. Ethics Statement

All samples were collected as part of standard clinical care, and all studies were approved by the Ethics Commission at the University of Lübeck and are in accordance with the Declaration of Helsinki (18-356). Patients gave written informed consent regarding routine diagnostic and academic assessment of their biopsy specimen as well as transfer of their clinical data.

### 4.9. Availability of Data and Materials

All processed sequencing data will be released into the immuneAccess database (Adaptive Biotechnologies, Seattle, WA, USA) in form of a dedicated project (“TCR repertoire in HGBL with MYC, BCL2 and/or BCL6 rearrangements”) upon publication of the manuscript.

## 5. Conclusions

In summary, our analysis reveals adverse prognostic implications of highly expanded intra-tumor TCR clonotypes in HGBL-DH/TH and delineates a clonal architecture of the tumor-infiltrating TCR repertoire distinct from tnDLBCL beyond merely age-related differences. Moreover, several entity-exclusive clonotypes, suggestive of tumor-neoantigen selection are identified and our data show that high baseline GPS correlates with elevated rates of relapse, refractory disease and overall survival. These opposing surrogate parameters of adaptive and innate immunity response constitute promising means of risk-stratification and treatment guidance requiring further validation.

## Figures and Tables

**Table 1:** Clinical characteristics of the study group.

Characteristics	DHL (n = 40)	THL (n = 7)	Triple-Negative DLBCL (n = 27)
**Age (yrs.; median + range)**	73.0 (35–89)	69.0 (42–79)	70 (18–87)
**Sex**			
Female	19 (47.5%)	2 (28.6%)	13 (48.1%)
Male	21 (52.5%)	5 (71.4%)	14 (51.9%)
**R-IPI**			
0	1 (2.5%)	-	1 (3.7%)
1–2	16 (40.0%)	2 (28.6%)	15 (55.6%)
>2	23 (57.5%)	5 (71.4%)	11 (40.7%)
**Stage (Ann Arbor)**			
I	7 (17.5%)	-	2 (7.4%)
II	8 (20.0%)	3 (42.8%)	11 (40.7%)
III	5 (12.5%)	2 (28.6%)	8 (29.6%)
IV	20 (50.0%)	2 (28.6%)	6 (22.2%)
**B-Symptoms**			
Yes	22 (55.0%)	4 (57.2%)	12 (44.4%)
No	18 (45.0%)	3 (42.8%)	15 (55.6%)
**Extranodal sites**			
0	10 (25.0%)	3 (42.8%)	10 (37.0%)
1–2	28 (70.0%)	4 (57.2%)	17 (63.0%)
>2	2 (5.0%)	-	-
**ECOG PS**			
0–2	37 (92.5%)	7 (100.0%)	24 (88.9%)
>2	3 (7.5%)	-	3 (11.1%)
**LDH**			
Normal	11 (27.5%)	1 (14.3%)	14 (51.9%)
Elevated	29 (72.5%)	6 (85.7%)	13 (48.1%)
**Glasgow prognostic score (GPS)**			
GPS 0	12 (30.0%)	1 (14.3%)	17 (63.0%)
GPS 1	8 (20.0%)	1 (14.3%)	7 (25.9%)
GPS 2	20 (50.0%)	5 (71.4%)	3 (11.1%)
**CNS involvement at diagnosis**			
Yes	1 (2.5%)	-	-
No	45 (97.5%)	7 (100.0%)	27 (100.0%)

DLBCL, diffuse large B-Cell Lymphoma; Yrs., years; CNS, central nervous system; LDH, Lactate dehydrogenase; ECOG; Eastern cooperative oncology group; PS, performance status.

**Table 2:** Treatment modalities of the study group.

Characteristics	DHL (n = 40)	THL (n = 7)	Triple-Negative DLBCL (n = 27)
**Frontline Therapy regimen**			
R-CHOP-like	27 (67.5%)	5 (71.4%)	23 (85.2%)
DR	7 (25.9%)	3 (60.0%)	10 (43.5%)
Others	12 (30.0%)	2 (28.6%)	3 (11.1%)
Refusal of treatment	1 (2.5%)	-	1 (3.7%)
**Response rates**			
CR	15 (37.5%)	1 (14.3%)	14 (51.9%)
PR	17 (42.5%)	3 (42.9%)	11 (40.7%)
SD	3 (7.5%)	1 (14.3%)	-
PD	5 (12.5%)	2 (28.6%)	2 (7.4%)
Relapse rate	21 (52.5%)	6 (85.7%)	4 (14.8%)
**2nd line treatment regimens (n = 35)**			
R-DHAP	7 (33.3%)	2 (33.3%)	1 (25.0%)
R-Bendamustine	3 (14.3%)	2 (33.3%)	1 (25.0%)
Auto/Allo HSCT	4 (19.4%)	1 (16.7%)	-
Others	5 (23.8%)	-	2 (50.0%)
Refusal of treatment	2 (9.5%)	1 (16.7%)	-
**Toxicity profile (1st line)**			
Cytopenia grade III/IV	13 (32.5%)	3 (42.9%)	11 (40.7%)
Polyneuropathy	5 (12.5%)	1 (14.3%)	7 (25.9%)
GI toxicity	2 (5.0%)	-	-
Mucositis	2 (5.0%)	2 (28.6%)	-
Pneumonia	3 (7.5%)	-	1 (3.7%)
Acute kidney injury	5 (12.5%)	-	-
Sepsis	12 (30.0%)	-	7 (25.9%)

DLBCL, diffuse large B-Cell Lymphoma; DR, dose-reduction; R, rituximab; CHOP, cyclophosphamide, doxorubicin, vincristine, prednisolone; Others, other rituximab-based regimen (e.g., R-Bendamustine), GMALL protocols or palliative cytoreductive treatment.

**Table 3:** Histopathological characteristics in the study group.

Characteristics	DHL (n = 40)	THL (n = 7)	Triple-Negative DLBCL (n = 27)
**Hans classifier and IHC**			
GCB	34 (85.0%)	6 (85.7%)	2 (7.4%)
Non-GCB	6 (15.0%)	1 (14.3%)	25 (92.6%)
BCL2	28 (70.0%)	5 (71.4%)	1 (3.7%)
BCL6	33 (82.5%)	6 (85.7%)	3 (11.1%)
MYC	25 (62.5%)	4 (57.1%)	-
CD10	31 (77.5%)	5 (71.4%)	3 (11.1%)
MUM1/IRF4	9 (22.5%)	2 (28.6%)	3 (11.1%)
Ki-67 (median, range)	85% (40–100%)	75% (50–95%)	85% (55–95%)
**T-cell infiltration**			
Percentage (median, range)	15% (1–30%)	10% (3–30%)	20% (5–30%)

DHL, double hit lymphoma; DLBCL, diffuse large B-Cell Lymphoma; IHC, immunohistochemistry; THL, triple hit lymphoma.

**Table 4:** Comparative TCR β sequencing of HGBL-DH/TH and tnDLBCL.

Analyzed Parameter	HGBL-DH/TH (n = 47)	tnDLBCL (n = 27)	*p*-Value
Productive TCR rearrangements	1504.5 (136–24,680)	3447 (164–21,745)	0.0050
Productive Clonality	0.036 (0.0009–0.3614)	0.08785 (0.0013–0.4437)	0.0427
% Maximum frequency clone	0.407 (0.205–1.822)	4.1884579 (0.4035117–44.8000014)	0.2473

**Table 5:** Major public T-Cell Receptor β CDR3 clonotypes found in HGBL-DH/TH but not tnDLBCL suggestive of a process of shared tumor-neoantigen selection within the tumor-infiltrating TCR repertoire, cross referenced with a publically available dataset.

Amino Acid	Present in HGBL-DH/TH	Mean Frequency (%)	Epitope Gene	Epitope SPECIES	V-Gene	J-Gene
CASSPLGETQYF	4	0.419	-	-	TCRBV18-01*01	TCRBJ02-05*01
CASRDTEAFF	4	1.822	-	-	TCRBV06	TCRBJ01-01*01
CASSPTRTGDYEQYF	3	1.593	-	-	TCRBV07-09	TCRBJ02-07*01
CASSDSSLTEAFF	3	0.201	EBNA3A	EBV	TCRBV06-04	TCRBJ01-01*01
CASSQGGSGEQFF	3	0.705	-	-	TCRBV04-03*01	TCRBJ02-01*01
CASSLGGSPEAFF	3	0.524	-	-	TCRBV07-02*01	TCRBJ01-01*01
CASSQGGSGELFF	3	0.468	-	-	TCRBV04-03*01	TCRBJ02-02*01
CASSLESGANVLTF	3	0.428	-	-	TCRBV07-06*01	TCRBJ02-06*01
CASSLGGAGANVLTF	3	0.395	-	-	TCRBV05-06*01	TCRBJ02-06*01
CASSLAGGGTEAFF	3	0.388	-	-	TCRBV28-01*01	TCRBJ01-01*01
CSARDRVSYNEQFF	3	0.302	-	-	TCRBV20	TCRBJ02-01*01
CSARDPGSSYEQYF	3	0.279	-	-	TCRBV20	TCRBJ02-07*01
CASSGTEAFF	3	0.216	-	-	TCRBV28-01*01	TCRBJ01-01*01
CSARSGNTEAFF	3	0.205	-	-	TCRBV20	TCRBJ01-01*01

## Data Availability

Data available in a publicly accessible repository (immuneAccess database -Adaptive biotechnologies) that does not issue DOIs (in form of a dedicated project: “TCR repertoire in HGBL with MYC, BCL2 and/or BCL6 rearrangements”).

## Supplementary Materials

The following are available online at https://www.mdpi.com/2072-6694/13/4/887/s1, Supplementary methods: Isolation of genomic DNA; Calculation of Shannon’s entropy; Comparative analysis with DLBCL and public data mining, Table S1: Clinical and histopathological characteristics of tnDLBCL, Table S2: Antibodies used; Figure S1: Further analysis of the clinical impact of the TCR repertoire clonality in HGBL-DH/TH; Figure S2: Correlation between productive TCR rearrangements and GPS; Figure S3: Correlation between the number of productive rearrangements and the number of quantitative T-cell infiltration.
