# Supplementary material for: Systemic Inflammation and Tumour-Infiltrating T-Cell Receptor Repertoire Diversity Are Predictive of Clinical Outcome in High-Grade B-Cell Lymphoma with MYC and BCL2 and/or BCL6 Rearrangements
Source: Cancers (Basel). 2021 Feb 20;13(4):887. doi: 10.3390/cancers13040887 (PMC7924187; doi:10.3390/cancers13040887)
Supplement: Supplementary file 1 [file cancers-13-00887-s001.pdf]

Supplementary Materials

Supplementary methods

Isolation of genomic DNA

Isolation of genomic DNA from FFPE sections was performed employing the QIAamp 250 DNA Mini Kit (Qiagen, Hilden, Germany) in accordance with manufacturer's instructions. Quantification and purity assessment was determined via photometric measurement (Nanodrop ND-1000, Thermo Scientific, Schwerte, Germany).

Calculation of Shannon's entropy (*H*)

Shannon's entropy was calculated based of the frequency of particular sequences. Said normalized entropy was employed as an established measure of diversity within the spectrum individual T-cell receptor repertoires measuring sample richness and the degree of unevenness in clone frequencies, as described (1). Further we calculated the reciprocal normalized entropy in order to describe clonality of "productive sequences" within the sample as a completely monoclonal composition (value = 1) to a perfectly polyclonal T-cell population with every TCR β chain represented once (value = 0). These calculations were performed according to these mathematical equations:

$$\text{Entropy} = H = -\sum_{i=1}^{N} \rho_i \log_2(\rho_i) \qquad \text{Clonality} = 1 - H/\log_2(\text{N})$$

Comparative analysis with DLBCL and public data mining

Both simultaneously as well as sequentially acquired samples were integrated into the comparative analysis, thus including all available samples from a given patient, if the predefined quality control standards were met. Primarily, clonotypes were categorized according to their distribution among cases and controls of the study. If a clonotype was shared between three or more patients, it was defined as "public".

Additionally, clonotypes were sequentially stratified according to their degree of expansion with an arbitrary cut-off between "major" and "minor" types at 0.2% average frequency and clones were filtered for exclusive presence in HGBL-DH/TH but not tnDLBCL. Clones only identified in multiple samples from one patient were excluded from subsequent analysis.

Shared tumor-neoantigen selection was suspected in all clonotypes meeting these predefined criteria within the tumor-infiltrating TCR repertoire of HGBL-DH/TH. Expanded clonotypes were additionally screened in the largest, clinically annotated, publically available TCR repertoire dataset comprised of healthy donors as well as a wide range of patients with various diseases (VDJdb) (2). Clonotypes, were annotated in accordance with previous descriptions in the context of other diseases. For comparative purposes, we included an analysis, which incorporated TCR sequencing data from non-diseased lymph node samples included in a recent study by Keane *et al.* (3). These were processed at Adaptive Biotechnologies while our samples were sequenced in Europe albeit using the same kits and hardware to the same specifications.

Supplementary Table S1. Clinical and histopathological characteristics of triple negative (FisH negative for aberrations at *cMYC*, *BCL2* and *BCL6*) DLBCL

Sample-ID	Age	Sex	Localisation	EBV (EBER)	Nodal/Extranodal	BCL2	BCL6	cMYC
tnDLBCL1	75	m	Stomach	pos	Extranodal	neg	neg	neg
tnDLBCL2	78	w	Spleen	pos	Extranodal	neg	neg	neg
tnDLBCL3	84	m	Skin	pos	Extranodal	neg	neg	neg
tnDLBCL4	70	m	Kidney	pos	Extranodal	neg	neg	neg
tnDLBCL5	77	w	LN	pos	Nodal	neg	neg	neg
tnDLBCL6	74	m	LN	pos	Nodal	neg	neg	neg
tnDLBCL7	60	w	LN	pos	Nodal	neg	neg	neg
tnDLBCL8	78	w	LN	pos	Nodal	neg	neg	neg
tnDLBCL9	70	w	LN	pos	Nodal	neg	neg	neg
tnDLBCL10	62	m	Tonsil	pos	Extranodal	neg	neg	neg
tnDLBCL11	68	w	Pancreas	neg	Extranodal	neg	neg	neg
tnDLBCL12	74	w	Mamma	neg	Extranodal	neg	neg	neg
tnDLBCL13	81	m	Bladder	neg	Extranodal	neg	neg	neg
tnDLBCL14	57	m	M. iliopsoas	neg	Extranodal	neg	neg	neg
tnDLBCL14.1	57	m	M. iliopsoas	neg	Extranodal	neg	neg	neg
tnDLBCL14.2	57	m	M. iliopsoas	neg	Extranodal	neg	neg	neg
tnDLBCL15	27	m	LN	neg	Nodal	neg	neg	neg
tnDLBCL16	65	m	Tonsil	neg	Extranodal	neg	neg	neg
tnDLBCL17	18	w	Tongue	neg	Extranodal	neg	neg	neg
tnDLBCL18	69	w	Palate	neg	Extranodal	neg	neg	neg
tnDLBCL19	57	w	LN	neg	Nodal	neg	neg	neg
tnDLBCL20	53	w	Tonsil	neg	Extranodal	neg	neg	neg
tnDLBCL20.1	89	w	Breast	neg	Extranodal	neg	neg	neg
tnDLBCL21	71	m	LN	neg	Nodal	neg	neg	neg
tnDLBCL21.1	71	m	LN	neg	Nodal	neg	neg	neg
tnDLBCL22	63	m	LN	neg	Nodal	neg	neg	neg
tnDLBCL22.1	63	m	LN	neg	Nodal	neg	neg	neg
tnDLBCL23	63	m	LN	neg	Nodal	neg	neg	neg
tnDLBCL24	73	m	LN	neg	Nodal	neg	neg	neg
tnDLBCL25	34	m	LN	neg	Nodal	neg	neg	neg
tnDLBCL26	77	w	LN	neg	Nodal	neg	neg	neg
tnDLBCL26.1	77	w	LN	neg	Nodal	neg	neg	neg
tnDLBCL27	37	m	LN	neg	Nodal	neg	neg	neg
tnDLBCL28	37	m	LN	neg	Nodal	neg	neg	neg

Supplementary Table S2. Antibodies used

Antibody	Supplier	Clone
Bcl2	Lab Vision	100/D5
Bcl6	Dako	BG-B6p
CD10	Menarini	56C6
MUM-1 (Irf4)	Dako	Mum 1P
Ki-67	Dako	Mib-1
MYC	Roche	Y69
CD4	Dako	4B12
CD8	Dako	C8/144B
CD20	Thermo Fisher	MA5-13141
CD3	Dako	Clone F7.2.38
B2M	Dako	Polyclonal
HLA-DR	Dako	Clone CR3/42

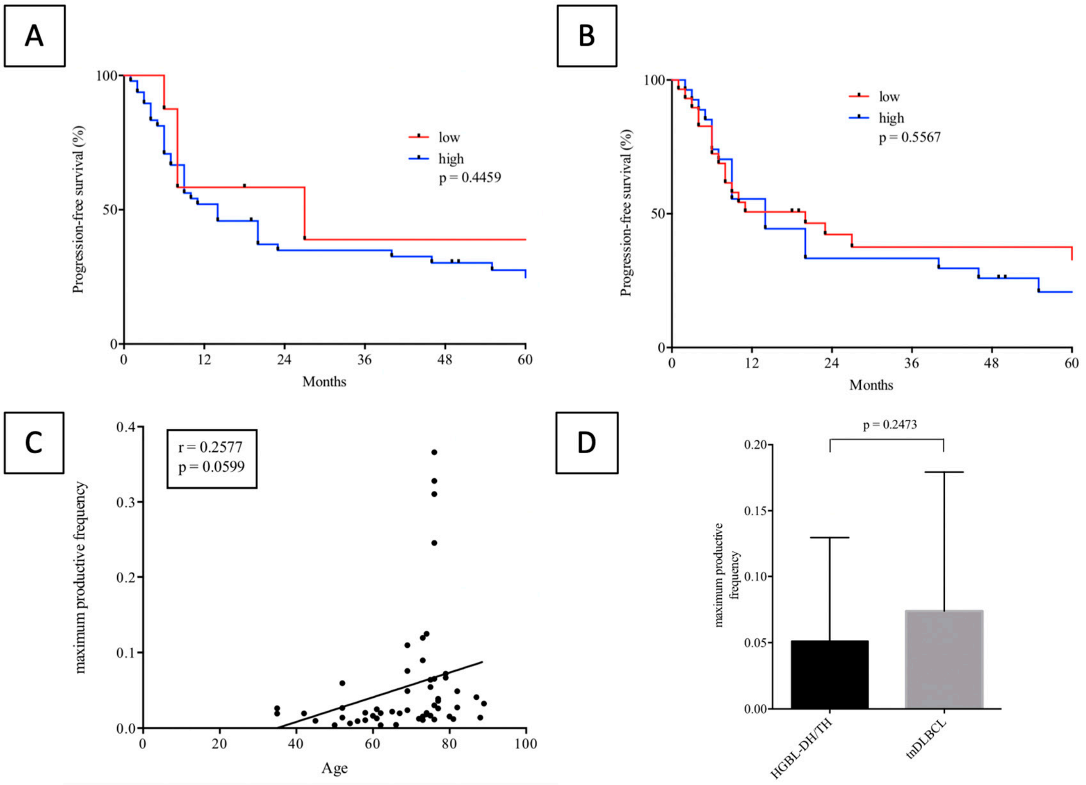

Supplementary Figure S1. Progression-free survival (PFS) (A, B) of HGBL-DH/TH patients stratified according to productive TCR clonality (A) and maximum productive frequency (B) does not display significant prognostic capabilities of the clonal architecture of the tumour-infiltrating TCR repertoire in HGBL-DH/TH with regard to PFS (productive clonality: p = 0.4459; maximum productive frequency: p = 0.5567). Maximum productive frequency in HGBL-DH/TH patients is bordering on statistical significance to be a significant function of age (p = 0.0599; r = 0.2577, C). In terms maximum productive frequency no significant difference was observed regarding clonality measures between HGBL-DH/TH and tnDLBCL upon comparative analysis (p = 0.2473, D).

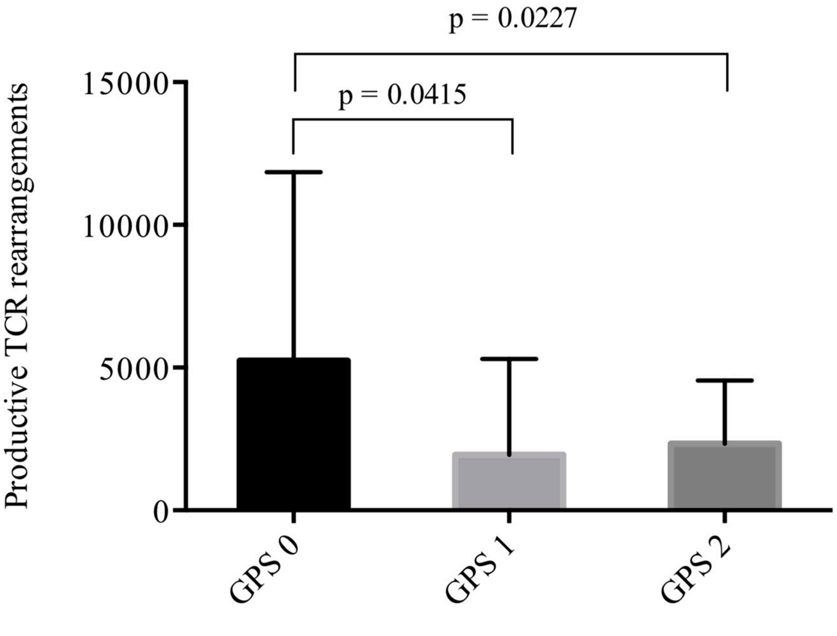

Supplementary Figure S2. A significant higher rate of productive TCR rearrangements could be detected in patients with aggressive B-cell lymphomas with a GPS of 0 compared to a GPS of 1 (p = 0.0415) or a GPS of 2 (p = 0.0227).

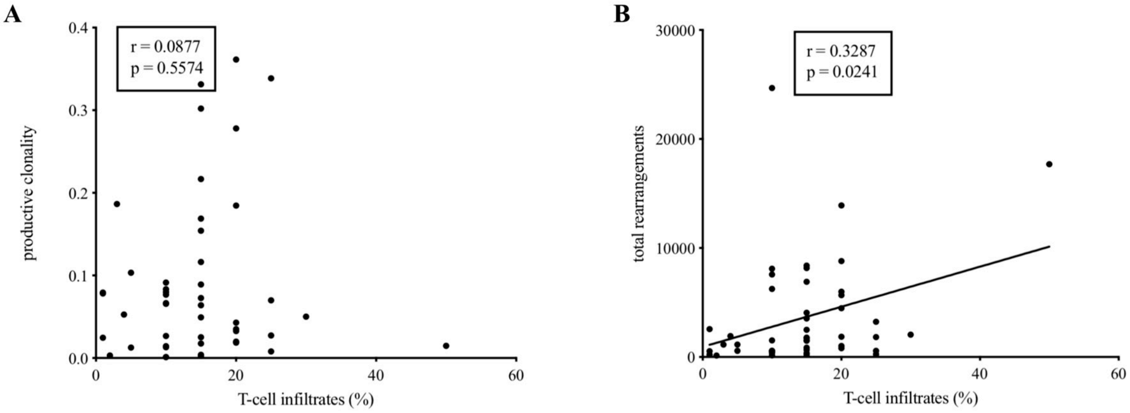

Supplementary Figure S3. In a given sample, there was statistically significant correlation between the number of productive rearrangements but not productive clonality and the number of quantitative T-cell infiltration.

Supplemental references

1. Robins HS, Campregher PV, Srivastava SK, et al. Comprehensive assessment of T-cell receptor beta-chain diversity in alphabeta T cells. Blood. 2009 Nov 5;114(19):4099-107. Epub 2009/08/27. doi:10.1182/blood-2009-04-217604. Cited in: Pubmed; PMID 19706884.

2. Shugay M, Bagaev DV, Zvyagin IV, et al. VDJdb: a curated database of T-cell receptor sequences with known antigen specificity. Nucleic Acids Res. 2018 Jan 4;46(D1):D419-D427. doi:10.1093/nar/gkx760. Cited in: Pubmed; PMID 28977646.

3. Keane, C.; Gould, C.; Jones, K.; Hamm, D.; Talaulikar, D.; Ellis, J.; Vari, F.; Birch, S.; Han, E.; Wood, P., et al. The T-cell Receptor Repertoire Influences the Tumor Microenvironment and Is Associated with Survival in Aggressive B-cell Lymphoma. *Clin Cancer Res* **2017**, *23*, 1820-1828, doi:10.1158/1078-0432.CCR-16-1576.
